# Supplementary material for: Cold-induced muscle atrophy in zebrafish: Insights from swimming activity and gene expression analysis
Source: Biochem Biophys Rep. 2023 Oct 31;36:101570. doi: 10.1016/j.bbrep.2023.101570 (PMC10641114; doi:10.1016/j.bbrep.2023.101570)
Supplement: Multimedia component 1 [file mmc1.docx]

| Table S1. Primer sequences for real-time PCR | | | |
| --- | --- | --- | --- |
| Primer name | Sequence | expected product size (bp) | gene ID |
| ef1alpha F | CAAGGAAGTCAGCGCATACA |  |  |
| ef2alpha R | TCTTCCATCCCTTGAACCAG | 134 | ENSDARG00000020850 |
| myhz2 F | GACGAGTGCTCTGAGCTGAA |  |  |
| myhz2 R | GGCATGTTTTTCCTTTTCCA | 78 | ENSDARG00000012944 |
| MuRF1 F | CCTGGCTTTGAGAGTATGGACC |  |  |
| MuRF1 R | GCCCCTTGCCTCACAGTTAT | 225 | ENSDARG00000028027 |
| Atrogin1 F | GAGCACCAAAGAGCGTCAT |  |  |
| Atrogin1 R | CACTCCACTCAGAGAAGGCAG | 155 | ENSDARG00000040277 |
| cblb F | CCGAGTGTAGTAAGTCTTTCGT |  |  |
| cblb R | TGGTTGTTGTCCAGAATAGGAG | 97 | ENSDARG00000015199 |
| nedd4 F | AAAACAGGCGATGCAAGGAC |  |  |
| nedd4 R | AGGCTCCAAAAACTCCCAAC | 74 | ENSDARG00000099843 |
| foxo1a F | CCCACACACATAATACAGGACT |  |  |
| foxo1a R | GAAAATATGAGGTGAAACGCGA | 162 | ENSDARG00000099555 |
| foxo1b F | TAGAAAGAGTCCTTCACGACAC |  |  |
| foxo1b R | GCAGATGACATGTCTATCCAGA | 141 | ENSDARG00000061549 |
| foxo3a F | CTGACCTTGCAGGAACTATGAA |  |  |
| foxo3a R | TCAAGCTGATGTTGTCCAGAAG | 81 | ENSDARG00000023058 |
| foxo3b F | AAGATTTGCACTAGTTGAAGCG |  |  |
| foxo3b R | CACTCCTCTTTACTGTCTTGGT | 118 | ENSDARG00000042904 |
