# Supplementary material for: Cold-induced muscle atrophy in zebrafish: Insights from swimming activity and gene expression analysis
Source: Biochem Biophys Rep. 2023 Oct 31;36:101570. doi: 10.1016/j.bbrep.2023.101570 (PMC10641114; doi:10.1016/j.bbrep.2023.101570)
Supplement: Multimedia component 2 [file mmc2.pptx]

## Slide 1
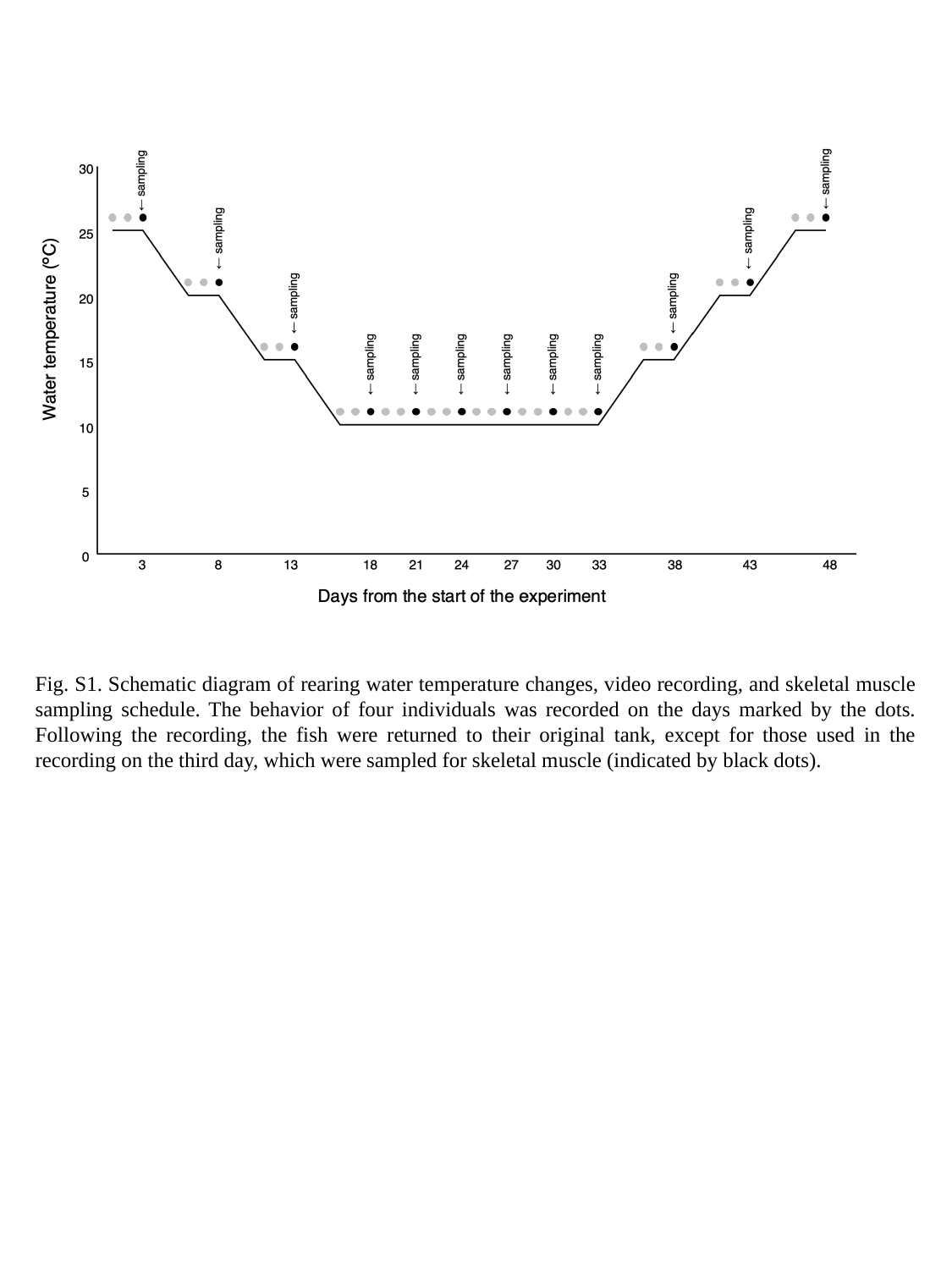

Fig. S1. Schematic diagram of rearing water temperature changes, video recording, and skeletal muscle sampling schedule. The behavior of four individuals was recorded on the days marked by the dots. Following the recording, the fish were returned to their original tank, except for those used in the recording on the third day, which were sampled for skeletal muscle (indicated by black dots).

## Slide 2
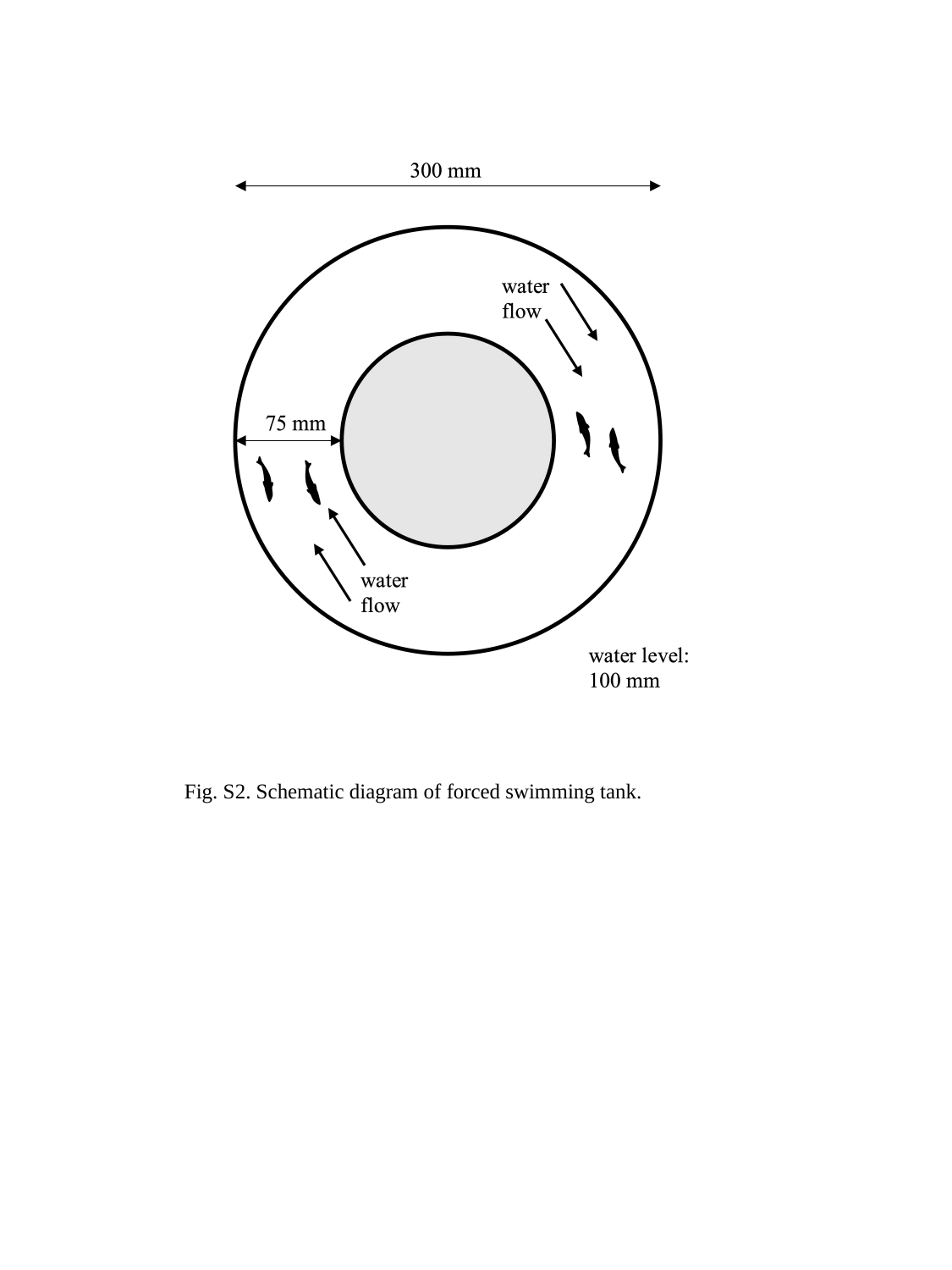

Fig. S2. Schematic diagram of forced swimming tank.

## Slide 3
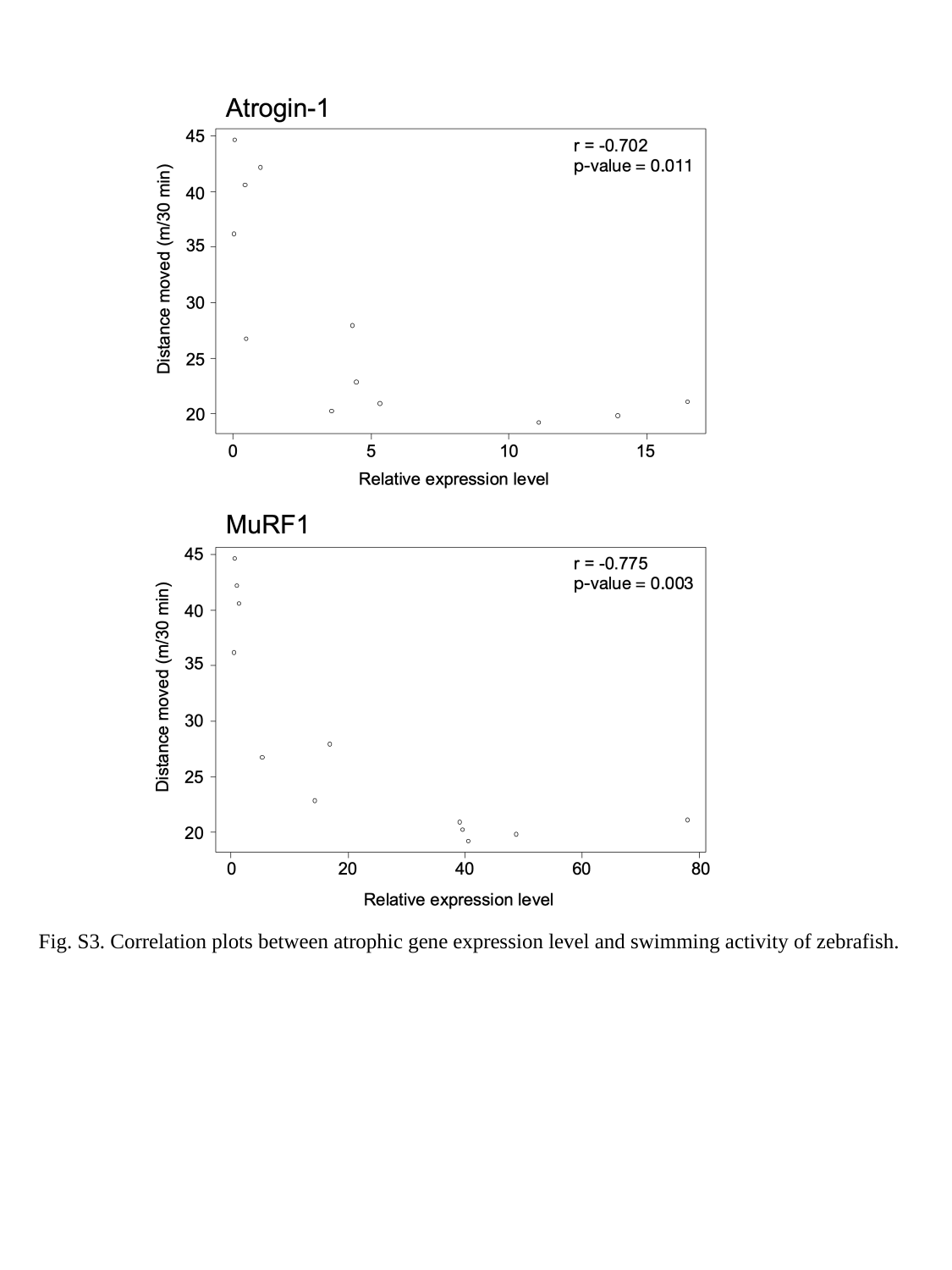

Fig. S3. Correlation plots between atrophic gene expression level and swimming activity of zebrafish.

## Slide 4
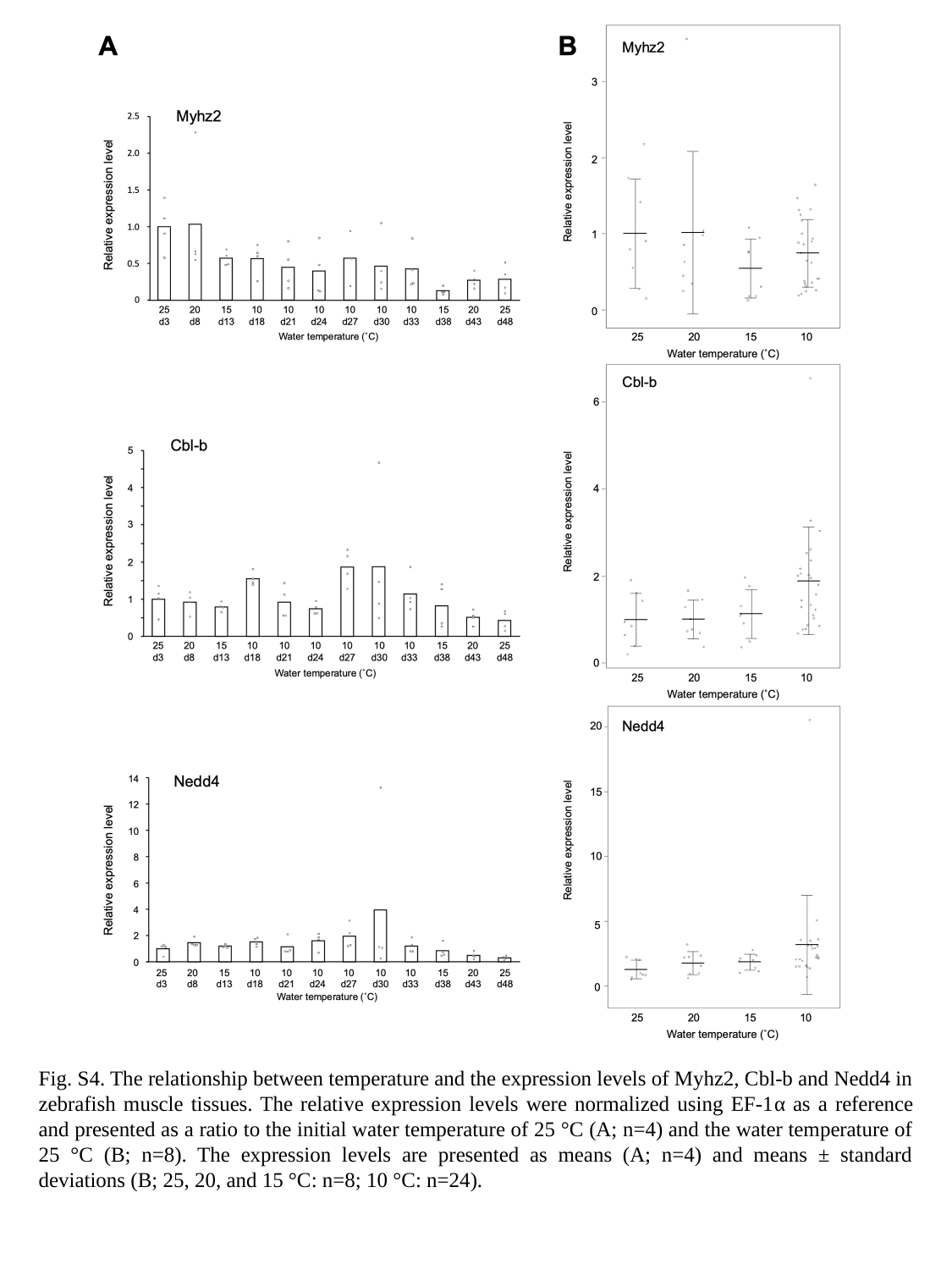

Fig. S4. The relationship between temperature and the expression levels of Myhz2, Cbl-b and Nedd4 in zebrafish muscle tissues. The relative expression levels were normalized using EF-1α as a reference and presented as a ratio to the initial water temperature of 25 °C (A; n=4) and the water temperature of 25 °C (B; n=8). The expression levels are presented as means (A; n=4) and means ± standard deviations (B; 25, 20, and 15 °C: n=8; 10 °C: n=24).

## Slide 5
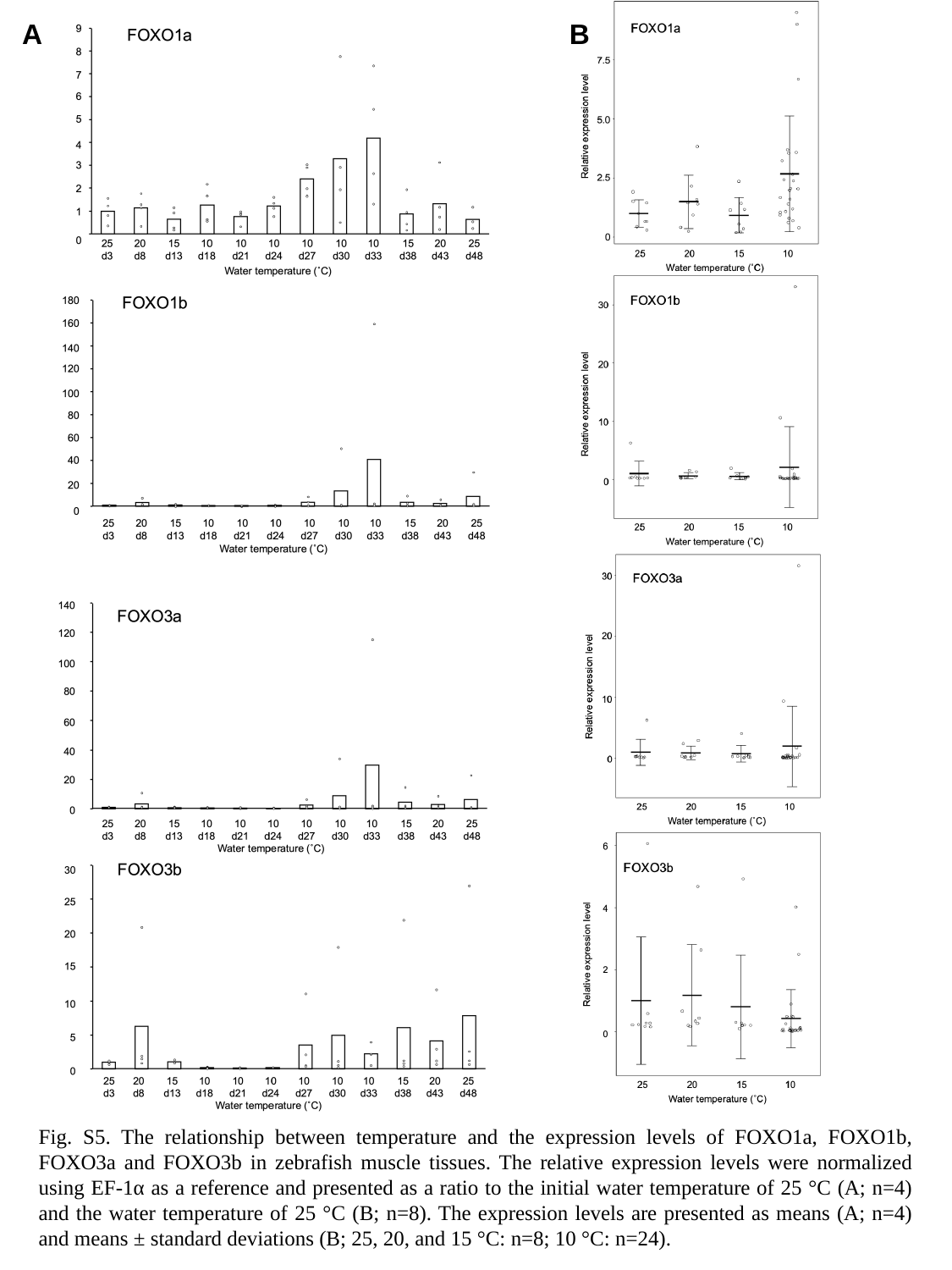

A
B
Fig. S5. The relationship between temperature and the expression levels of FOXO1a, FOXO1b, FOXO3a and FOXO3b in zebrafish muscle tissues. The relative expression levels were normalized using EF-1α as a reference and presented as a ratio to the initial water temperature of 25 °C (A; n=4) and the water temperature of 25 °C (B; n=8). The expression levels are presented as means (A; n=4) and means ± standard deviations (B; 25, 20, and 15 °C: n=8; 10 °C: n=24).

## Slide 6
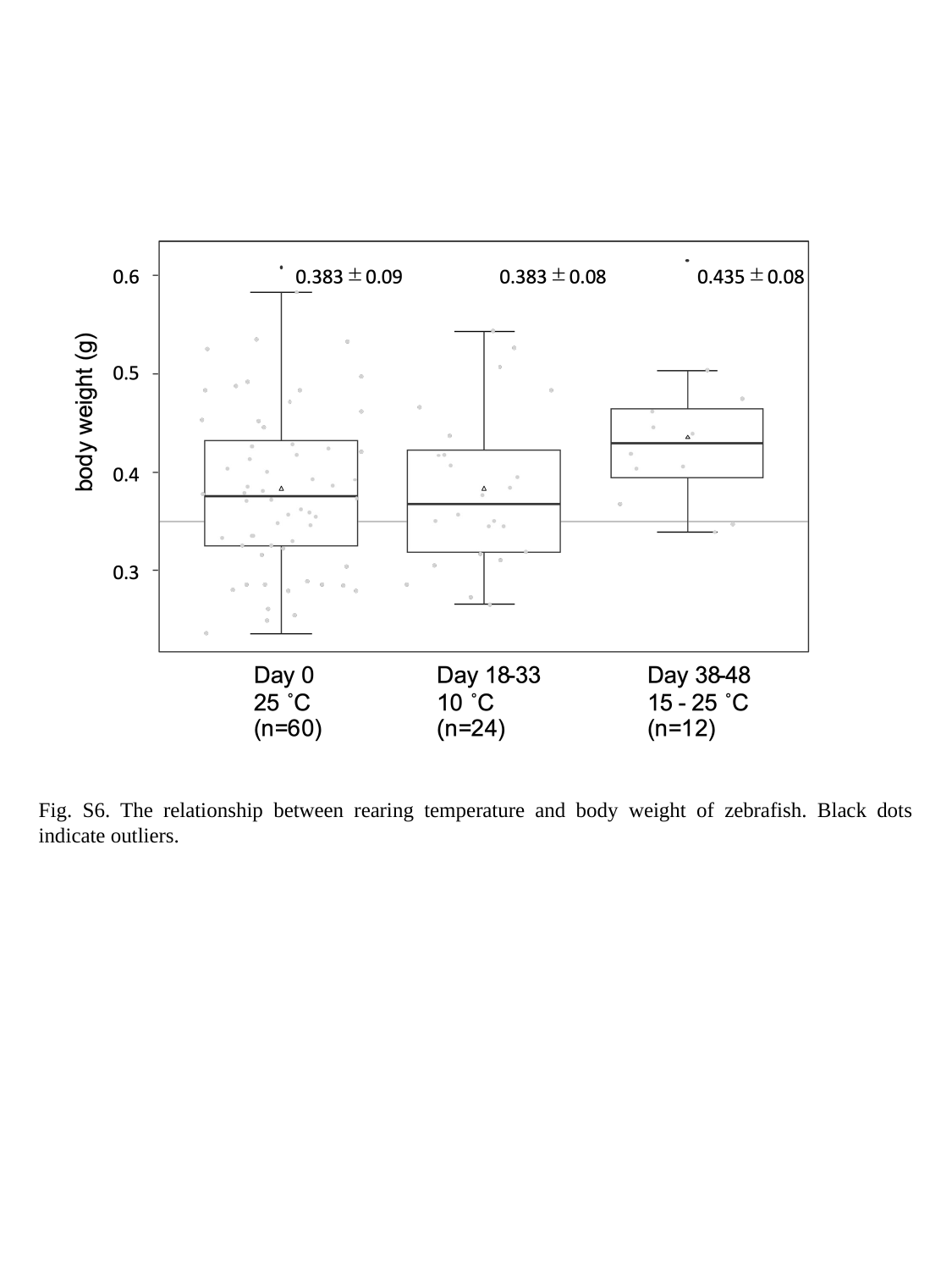

Fig. S6. The relationship between rearing temperature and body weight of zebrafish. Black dots indicate outliers.
